# Enhancing capabilities in health professions education

**DOI:** 10.5116/ijme.5641.060c

**Published:** 2015-11-22

**Authors:** Sandra E. Carr, Susan J. Miller, Zarrin S. Siddiqui, Diana R.D. Jonas-Dwyer

**Affiliations:** 1 Faculty of Medicine, Dentistry and Health Sciences, Education Centre, The University of Western Australia, Australia

**Keywords:** Faculty development, postgraduate, health professions

## Abstract

**Objectives:**

This article documents the results of ongoing summative program evaluation of a suite of postgraduate courses at The University of Western Australia designed to enhance the educational capabilities, academic leadership and scholarly output of health professionals.

**Methods:**

Commencing students were invited to participate in this descriptive, longitudinal study that surveyed students at commencement and subsequently over a seven year period. Data was collected at baseline and follow-up in relation to the respondents’ educational leadership responsibilities, promotions, involvement in new educational programs, and recognition for contributions towards student learning, educational scholarly outputs and involvement in training programs.

**Results:**

The respondents came from a wide range of health professions and worked in various roles, with a quarter already holding leadership positions. During the follow-up period, half reported receiving a new promotion or moving to new positions requiring educational leadership. Those identifying as being involved with the development of new educational programs doubled and 34% received a new teaching award. Scholarly productivity doubled with 45% giving an oral presentation related to education, 21% publishing and 29% being successful in obtaining funding related to an education project.

**Conclusions:**

These postgraduate courses in health professions education appear to be positively influencing graduates’ capabilities, especially in the areas of educational leadership skills and scholarly productivity. For those looking to develop a community of leaders in health professions education, the authors offer some suggestions.

## Introduction

In the area of health professions education (HPE), most academic appointments are made based on how knowledgeable the academic is in their discipline or on their research abilities, over educational expertise. With the increasing numbers of health-related professional programs, there are limited numbers of staff with teaching qualifications to meet the educational needs of tertiary institutions and teaching hospitals both locally in Western Australia (WA), nationally and internationally. This lack of qualified teaching staff has the potential to influence student learning experiences and their attainment of the required learning outcomes.^1^^-^^3^ This in turn can affect the outcomes for quality patient care and safety.^4^

In their review of faculty development initiatives, Steinert et al. suggested that due to the increasing complexity of medical education and practice, leadership roles have become more important.^5^ Others have also indicated that leadership development has been recognised as a critical component of faculty development in health professions education; along with teaching skills.^6^

In 2006, Gruppen et al. reviewed nine educational fellowship programs and reported that these programs documented gains in participant promotions, new leadership positions and scholarly productivity.^7^ The educational benefits included the creation of a pool of leaders and a community of knowledgeable scholars who serve as a catalyst for educational improvement across institutions. All of the programs reviewed emphasised more than just developing teaching skills.

Evaluation is an essential component for assessing the effectiveness of coursework programs. For many years, educators have used Kirkpatrick’s four levels of evaluation to determine the effectiveness of training courses.^8^ Level 1 is a measure of how participants feel about the aspects of a training program and Level 2 assesses the knowledge or skills acquired. Level 3 considers the extent to which participants change their behaviour; for example becoming educational leaders in their workplace, and Level 4 is a measure of final outcomes, such as increased scholarly output and practice as a teacher.

Gruppen et al. reported that the majority of evaluations of Faculty Development programs were short-term and focused on participants’ satisfaction and self-efficacy; Level 1 and 2 in Kirkpatrick’s model.^1^ Moreover, it has been suggested that researchers employ more rigorous and systematic research designs to evaluate the influence of faculty development, and to evaluate change over time in order to understand how leadership develops.^5^ Correspondingly, they found that changes in organisational practice such as educational scholarship and the establishment of collegial frameworks were not frequently examined.

In response to this need for health professionals to know how to teach, there have been a growing number of programs designed to equip health professionals with the required educational expertise around the world. Currently, five Australian universities offer a graduate certificate program in education for health professionals with some of these articulating to a Masters’ degree. In 2007, the Faculty of Medicine, Dentistry and Health Sciences Education Centre, commenced a suite of postgraduate courses (Graduate Certificate, Graduate Diploma, Masters by coursework or by research) in health professions education. It was anticipated that the courses would result in a community of health professions educators in WA with enhanced educational leadership skills and an increase in their educational scholarly output.

At The University of Western Australia (UWA) program evaluation at Levels 1 and 2 are measured using university course evaluations and summative assessments of student performance during the course. This study determined to evaluate the HPE courses at Level 3 and 4 of Kirkpatrick’s evaluation model, by exploring the influence of the health professions education courses at UWA on graduate capabilities, especially in the areas of developing educational leadership skills and scholarly productivity. Specifically the study sort to answer what were graduates aggregated increase in accomplishments and subsequent education related activity once completing the HPE course? For instance, were graduates obtaining more recognition for their teaching? Were they attaining new jobs or promotions as educators? Were they more involved with course design and evaluation? Were they contributing to the scholarship of education? Were they being seen as leaders in education within their workplace?

## Methods

### Program outline

The learning outcomes of the three core units of the HPE courses relate to adult learning educational principles; learning theories; strategies to facilitate clinical teaching and learning; different teaching approaches and learning styles; critical self-reflection skills; educational leadership; assessment and measurement; program evaluation; essential research design methods applied in HPE including data analysis techniques and critique of relevant HPE literature. Elective units include topics such as simulation, interprofessional education, clinical teaching and supervision, and other contemporary issues in health professions education. The Masters’ by research projects which are conducted by thesis or dissertation are based on the students’ own areas of interest and are generally related to their current roles in the workplace.

### Participants

All students who commenced the postgraduate courses in health professions education at the UWA between 2007 and 2014 were invited to participate in this longitudinal descriptive study (N=171). Participants were enrolled in any of the postgraduate courses (either part time or full time) and were studying in either face-to-face or online modes. The study population consisted of health professionals predominantly from Nursing, Midwifery and Medicine backgrounds (75%) with fewer from a range of other health professions; ages ranging from 21 to 61 years, with approximately 70% female and 30% male. All of those who agreed to participate completed a written consent form and the study obtained ethics approval from the UWA Human Ethics Research Office (RA/4/1/1746).

### Outcome variables of interest

The outcome variables of interest were:

   •   Promotions – in positions related to education

   •   Awards – recognised for excellence in teaching practice

   •   Practice – increased teaching and or course design practice

   •   Leadership – in the area of education

   •   Scholarly output via publications or conference presentations.

### Data collection methods

A survey that was based on similar research in the literature was developed locally.^1^ Items were constructed to collect information from the participants in relation to the outcome variables of interest; i.e. their educational leadership responsibilities, involvement in the development of new educational programs and whether they had received any recognition for their contribution towards student learning. They were also asked to detail their educational scholarly outputs by way of journal publications, conference presentations and educational grants. Finally, the respondents were asked to identify their experience as trainers; i.e. skill development of peers or colleagues in the area of education, and their participation in educational professional development training. The demographic data collected included age, gender, work area, work fraction, job title, length of time in current position and any promotions in the previous year. The researchers piloted the survey with four academic staff to enhance the face validity and changes were made based on their feedback.

### Procedures

Every student that consented to be included in the study was asked to complete the survey in the face-to-face course orientation session. Participants were then mailed the follow-up survey in 2009, 2011 and 2014. Descriptive statistics were applied to the demographic and categorical data, and are reported as percentages. The summarised results are presented for the baseline data and the aggregated follow-up data from respondents against the main outcome variables of interest. A thematic analysis of the content of the open-ended responses was conducted with the common recurring themes presented.

## Results

### Demographics

Eighty one students provided baseline data from 2007 to 2014 which represents 47% of the study population. The mean age of the respondents was 39 years (range 21-61 years) and 15% were male. They reported backgrounds in a wide range of professions, with the majority working in nursing (32%) and medicine (27%), followed by midwifery (17%) and physiotherapy (9%). The remainder worked in areas such as occupational therapy, radiography, podiatry and speech pathology. Two thirds of the participants were employed full-time and 73% had been in their current job for more than a year.

### Baseline data

The respondents reported working in a wide variety of positions and roles within their organisations. The four common representative themes identified were 1) those who teach formally in an educational institution; such as a lecturer, unit coordinator, medical educator and clinical coordinator, 2) health professionals who teach predominately at the bedside in hospitals; such as a medical registrar, consultant, radiologist, family physician, speech pathologist, occupational therapist, midwife, physiotherapist and registered nurse, 3) staff development trainers; such as staff development nurse, clinical supervisor/facilitator, training and staff developer and professional education coordinator, and 4) administrative roles such as Deputy Director, Project Officer and Project Coordinator.

Almost 63% reported being were responsible for providing training to others at either a local, national or international level with 50% of the training being delivered at their own institution. Additionally, 50% reported being involved with the development of new educational programs. These included programs such as simulation training, problem-based learning, communication skills training, portfolio writing and clinical skills in the ward environment. Others reported being involved in processes such as curriculum review, moving a hospital-based course to a university-based program, revision of a graduate program, developing student supervision packages, and planning an allied health induction and orientation program.

At the beginning of their postgraduate course, a 25% of respondents reported holding a leadership position such as supervising education, chairing educational committees, providing fellowship training, advising on accreditation issues, coordinating the provision of clinical supervision and placements, and being responsible for clinical learning of new graduates. Others held titles such as Director of Staff Development, Head of Staff and Student professional development, Simulation Course Director and Training Supervisor.

### Aggregated outcomes from follow-up

Thirty eight (47%) of the consented participants completed at least one follow-up survey with 10 (26%) completing all three follow-up surveys (2008, 2011, 2014). As presented in Table 1, 45% of respondents received a new promotion during the follow-up period and 50% stated they were now in new positions requiring educational leadership. There were now 70% who identified themselves as being involved with the development of new educational programs and 34% whom had received a new teaching award during the follow-up period. Examples of awards included a preceptor award, Graduate Certificate Tertiary Teaching Scholarship, Nurses Board Excellence Award nomination and hospital-based recognition for teaching. In regard to research output, 45% reported giving an oral presentation related to education at a conference during the follow-up period with 21 % having published and 29% being successful in obtaining funding related to an education project during follow-up.

**Table 1:** Summary of scholarly productivity outcomes at baseline and follow-up

Outcome variable of interest	Baseline (N=81) Count (%)	Aggregated follow- up (N=38) Count (%)
Promotion	21 (26)	17 (45)
Role in Educational Leadership	18 (22)	19 (50)
Developing new educational programs	42 (52)	27 (71)
Educational Awards	8 (10)	13 (34)
Peer reviewed publications in education	1 (1.2)	5 (13)
Non peer reviewed publications in education	5 (6)	3 (8)
Oral presentations on Education at Conferences	8 (13)	17 (45)
Educational Grants applied for (local, national, international	11 (13)	13 (34)
Educational Grants received (local, national, international	6 (7) (5 local,1 international)	11 (29) (9 local, 2 national)

## Conclusions

This study evaluated the scholarly productivity outcomes of students entering a postgraduate course in health professions education and explored graduates aggregated increase in accomplishments, and subsequent education-related activity once having completed the course.

It is recognised that a quarter of students beginning these postgraduate courses had been in a position requiring leadership in the area of education and half had been involved with the development of new educational programs without any relevant qualifications. This may have been the motivating factor for them to enrol in a postgraduate course.

The results of this study indicate that the courses in Health Professions Education at UWA have been successful in achieving the original aims of implementation, i.e. developing well-skilled teachers who are also educational leaders with increased scholarly output in education research. Of the participants that completed the survey over an extended period, nearly half received a promotion in their workplace. In addition, almost one fifth of these received an award or incentive related to teaching. Furthermore, educational scholarly research in terms of presenting at conferences publishing in journals and receiving an educational research grant was relatively high, while the students were completing their studies or after graduation.

The benefits of enhancing the skills of health professions educators have had an effect within and beyond the university, in health care institutions such as teaching hospitals. Although it is recognised that there would be factors other than participating in the HPE courses that may have contributed to the receiving of a promotion to a leadership position or increasing educational scholarly research output, it appears that a community of leaders in health professions education is emerging across the health care sector in WA.

Although, this work primarily informs the University of the effectiveness of the courses, and there are some limitations of generalising the findings due to relatively small numbers, another outcome of this evaluation is to make recommendations for others involved with enhancing health professions educators in these two areas, with the aim of building communities of leaders in the field of HPE. Based on the information collected in the current study, we have developed several suggestions for those looking to develop a community of leaders and scholars in health professions education in their university or workplace.

### 1. Deliver the courses/programs over an extended timeframe

The HPE courses at UWA range from a four month semester to one and a half years full-time. Gruppen et al.^1^ suggested that if a course is designed to promote educational leadership, short-term workshops may be unsuccessful because of their narrow scope. The authors stated that programs that extended over one to two years allowed for a more in-depth and integrated understanding of educational principles and practices. Steinert et al.^5^ also support this notion, recommending that the value of extended programs be explored when developing training in leadership development in medical education.

### 2.Include a diversity of participants

The students undertaking the UWA courses came from a wide range of health professions and fulfilled a variety of roles as teachers, staff development trainers and educational administrators. One of the strengths of the educational programs that Gruppen^7^ reviewed was found to be their interdisciplinary nature. The majority of programs described in the literature that are designed to develop teaching skills, leadership abilities and/or to increase scholarly output in education research are based in universities that implement faculty development programs for their own staff members.^9^^-^^16^ Gruppen et al^7^ suggested that successful programs firstly develop a community of educators that can be extended institution-wide thereby creating a community of leaders.

The FMDHS provide scholarships for current UWA staff within the Faculty to complete the HPE courses. In addition to offering the courses to staff within the university, we deliver the courses to health professionals from external institutions, such as hospitals and private practice. This adds to the diversity of the student population, thereby enhancing the opportunities for interprofessional learning amongst academics and health professionals, and as well as providing opportunities for exchange of ideas around current educational issues in the workplace.

### 3. Develop well-designed curricula

The structure of the HPE curriculum at UWA has clear learning outcomes, assessments that are relevant and aligned with the learning outcomes, and a wide range of collaborative teaching strategies based on adult learning principles. The student feedback regarding the quality and relevancy of the courses has been positive. This supports the findings of Gruppen,^7^ whose authors found that educational programs with a clear set of goals and objectives for the curriculum, and active student-centred learning were the most successful.

### 4. Enhance opportunities for leadership discussions/research collaborations

The authors of a program outlined in the literature identified several structural features that contributed to their ability to successfully achieve one of its objectives of developing an infrastructure that supports sustained faculty development. These included implementing organisational changes that supports faculty development and aligning the programs’ learning outcomes with their workplace requirements.^17^ This could take the form of providing opportunities for staff to attend ‘journal clubs’ or meet with other like-minded staff to set up research collaborations. At UWA, we conduct meetings with current and past Masters’ students to share research ideas and discuss possible opportunities for research collaborations. The flow-on effect is the development of individuals as educational leaders.

### 5. Enhance level of institutional support

“Faculty development has a role to play in nurturing and sustaining health professionals as teachers and educators, leaders and managers, and researchers and scholars. It can also help to enhance academic and career development as well as organizational change”.^18^ In a previous study, we found that lack of protected time, funding and institutional support were the most common reason for medical doctors not attending educational continuing professional development.^19^ Support for individuals, either by way of finances or protected time, in order for staff to undertake professional development as educators, apply for grants or attend education-related conferences contribute to the success of any program.^5^

### 6. Recognition for teaching-related activities

Some universities and health care institutions present awards for excellence in teaching. Others provide recognition for positive educational outcomes. Further acknowledgement that could be provided include the provision of resources (e.g. protected time and funding) and/or scholarships for undertaking educational professional development.

The area of health professions education is characterised by rapid change, due to the increasing demand for trained health care professionals.^20^ This article has provided some evidence that educational leadership and scholarly output are enhanced through the participation in university courses in health professions education. In addition, several practical formal and informal strategies have been suggested in order to enhance the quality of HPE, and ultimately the level of patient care and safety.

### Acknowledgments

The authors would like to acknowledge the teaching team engaged to deliver these courses in health professions education. In particular, we thank Dr’s Gabrielle Brand and Annette Mercer, and Ms Caroline Martin.

### Conflict of Interest

The authors declare that they have no conflict of interest.
